# Combination quetiapine therapy in the long-term treatment of patients with bipolar I disorder

**DOI:** 10.1186/1745-0179-1-7

**Published:** 2005-07-18

**Authors:** MC Hardoy, Alessandra Garofalo, Bernardo Carpiniello, JR Calabrese, MG Carta

**Affiliations:** 1 Division of Psychiatry, Department of Public Health, University of Cagliari, Italy; 2 University Hospitals of Cleveland, Cleveland, Ohio, USA

**Keywords:** Quetiapine, bipolar I disorder, mood disorders, long-term, combination, atypical antipsychotics

## Abstract

**Objective:**

Determine the long-term effectiveness of quetiapine in combination with standard treatments in preventing relapses for patients with bipolar I disorders

**Method:**

Twenty-one outpatients with type I bipolar disorder who had inadequate responses to ongoing standard therapies were treated with add-on quetiapine in an open-label study. The quetiapine dose was increased until clinical response occurred. Illness response was assessed using the Clinical Global Impression (CGI) scale. Relapse rates before and during quetiapine treatment were compared by calculating incidence risk ratios.

**Results:**

Quetiapine was added to ongoing standard therapy for 26 to 78 weeks. Thirteen patients received combination therapy for at least 52 weeks. The mean quetiapine dose received was 518 ± 244 mg/day. There were highly significant improvements in overall relapse rate, manic/mixed relapse rate, and depression relapse rate in the period during quetiapine treatment compared with the period before quetiapine was initiated. The calculated relative risk of relapse in the absence of quetiapine treatment was 2.9 overall (95% confidence interval, 1.5~5.6), 3.3 for manic/mixed relapse (95% confidence interval, 1.5~7.1), and 2.4 for depressive relapse (95% confidence interval, 1.3~4.4). The mean Clinical Global Impression scores improved significantly from baseline during 26 weeks of quetiapine treatment in 21 patients (p = 0.002) and remained significantly better during a 52-week treatment period in 13 patients (p = 0.036).

**Conclusion:**

Long-term treatment with quetiapine combination therapy reduced the probability of manic/mixed and depressive relapses and improved symptoms in patients with bipolar I disorder who had inadequate responses to ongoing standard treatment.

## Introduction

Type I bipolar disorder is a severe and usually chronic mood disorder that causes significant illness and life disruption and is characterized by recurring episodes of mania and depression.

The goals of pharmacological treatment for bipolar disorder are to stabilize mood and achieve remission from manic and depressive episodes and, after completing an acute phase of treatment, maintain remission and prevent the recurrence of episodes. In practice, these goals are often achieved by treatment with traditional mood-stabilizing agents, such as lithium, valproate, and/or lamotrigine, with addition of an antipsychotic or other psychotropic agents [1]. In randomized, placebo-controlled trials, lithium has been observed to reduce the risk of relapse by 41% and increase the time to requirement of additional medication for a manic or depressive episode (median survival time of 170 days versus 93 days for placebo [2-4]. When compared with placebo, lamotrigine also increased the time to intervention for a manic or depressive episode (median survival time of 200 days versus 93 days), but was notable for its superiority to lithium in preventing depressive relapse (57% versus 46% of patients were intervention-free for depression over 1 year [4]. Ideally, the risk of drug interactions, adverse events, and potential benefit for the individual patient must be evaluated for each additional medication and combination.

Although many combinations of these therapies are in common usage, longitudinal studies of patients with bipolar disorder in the community indicate that a large proportion of individuals remain ill or have serious symptoms for significant periods of time, despite treatment with several different medications during the course of the studies [5-8]. These observations suggest there is a continuing need for systematic investigations of therapeutic options for patients, particularly those with refractory disease.

Quetiapine has been shown to be effective either as combination therapy or monotherapy for the treatment of bipolar mania [9-11], and as monotherapy for the treatment of bipolar depression [12]. These studies have demonstrated that even in the absence of psychotic symptoms, patients benefit from quetiapine therapy during mania and depression. In general, atypical agents such as quetiapine are recommended for patients with bipolar disorder over conventional antipsychotic agents due to the more benign side-effect profile associated with atypicals [1].

In open-label trials, quetiapine has been found to reduce symptoms, with a well-tolerated side-effect profile for patients with poorly controlled bipolar and schizoaffective mood disorders [13], as well as rapid-cycling type I bipolar disorder [14]. These studies were carried out for 12 to 20 weeks with patients whose previous dose of mood-stabilizing medication was either discontinued or maintained. Large, randomized, clinical studies demonstrated the efficacy and tolerability of quetiapine as short-term (3 months) monotherapy and combination therapy during episodes of mania [11,15,16]. However, the effects of longer-term therapy with quetiapine for patients with refractory disease are less well understood. This study was designed to determine the long-term clinical efficacy of adding quetiapine to ongoing medications in patients who were responding inadequately to these medications. Symptomatology and relapse rates were evaluated during combination therapy for 26 to 78 weeks.

## Method

### Patients

For participation in the study, patients had to be 18 years of age or older and had to fulfill the DSM-IV diagnostic criteria for bipolar I disorder [17]. Exclusion criteria included pregnancy, breastfeeding in women, and a recent history of alcohol or drug abuse in both men and women. Consent for the study was obtained after giving patients a complete description of the study. Inadequate prior response to previous therapies was defined as the patient having failed to respond to various treatment strategies, such as lithium, including substitution or augmentation with other mood stabilizers and antipsychotics.

### Quetiapine dosing

Quetiapine was introduced at an initial dose of 25 mg during ongoing treatment with each patient's psychotropic medication (mood stabilizers, typical and atypical antipsychotics other than quetiapine; Table 1). The dose was increased from 25 mg per day for the first 2 days to 50 mg for 2 more days, and then adding 50 mg every 2 days until a clinical response was achieved. This dose was maintained for the duration of the study. Patients were seen every 15 days (or more frequently if needed) to assess response and side effects, to record adherence to medication, and to adjust doses as necessary. Patients were rated by the same clinician throughout the study period. Patients could be withdrawn from participation in the study at their request or on the basis of clinical judgment or abnormal safety assessments.

**Table 1:** Patients' ongoing medication

Medication	Number of patients
Carbamazepine	1
Chlorpromazine	2
Clozapine	2
Gabapentin	1
Haloperidol	5
Lithium	13
Olanzapine	5
Risperidone	2
Sodium valproate	2

Psychopathologic evaluation and severity assessments were performed at baseline and at every visit, including an overall opinion in several areas: seriousness of the illness, general improvement, rate of therapeutic efficacy, and assessment of various psychiatric symptoms.

### Calculation of relative risk of relapse

The number of relapse events per patient-years of treatment were calculated and compared for the total patient population (N = 21) during the period 12 to 38 weeks before beginning quetiapine and during the period of quetiapine treatment. The following clinical events were included in the calculation of relapse rates before and during treatment with quetiapine: rehospitalization at the discretion of the patient's attending physician, any treatment in a day hospital or clinic, and an increase of 1 or more in Clinical Global Impression (CGI) score that was accompanied by a change in therapy.

### Clinical Global Impression

Clinical response was evaluated using the CGI scale [18], which was administered to all patients at the beginning of the follow-up and at 12 and 26 weeks. In a subgroup of patients who completed 52 weeks of treatment, a comparison was made between the baseline and Week 52 CGI scores.

### Statistical analysis

Confidence intervals to compare the relative risk of relapse were calculated using the simplified method of Miettinen [19]. Mean CGI scores were compared using a one-way analysis of variance (ANOVA) test.

## Results

### Patients

Twenty-one patients (14 men, 7 women; mean age 40.7 ± 12.7 years) who were receiving various medications for type I bipolar disorder, were treated with quetiapine in combination with their previous medication (Table 1). The final dose of quetiapine (mean 518 mg/day ± 244 mg/day) was maintained for 26 to 78 weeks. Thirteen patients received combination therapy for more than 52 weeks.

### Efficacy

**Figure 1:**
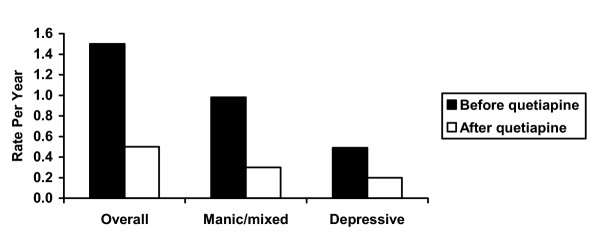
Rate of overall, manic/mixed, and depressive relapses/year before and after the addition of quetiapine to ongoing therapy.

A statistically significant decrease in overall relapse rate was observed during 26 to 78 weeks of quetiapine therapy compared with the 12 months previous to the introduction of quetiapine, from 12 relapse events per 8.1 person-years (1.5) to 10 relapse events per 19.5 person-years (0.5) (Fig. 1). The relative risk of relapse in the absence of quetiapine therapy was 2.9 (95% confidence interval, 1.5~5.6).

Similarly, the rates of manic/mixed relapse and depressive relapse were significantly decreased during the 26- to 78-week period of quetiapine treatment (Fig. 1). Manic or mixed relapses occurred at a rate of 8 events per 8.1 person-years (0.98) in the 12 months before quetiapine therapy compared with 6 events per 19.5 person-years (0.3) during quetiapine therapy, for a relative risk of relapse of 3.3 (95% CI, 1.5~7.1). Depressive relapses occurred at a rate of 4 events per 8.1 person-years (0.49) before initiation of quetiapine therapy versus 4 events per 19.5 person-years (0.2) during quetiapine therapy, for a relative risk of relapse of 2.4 (95% CI, 1.3~4.4).

**Figure 2:**
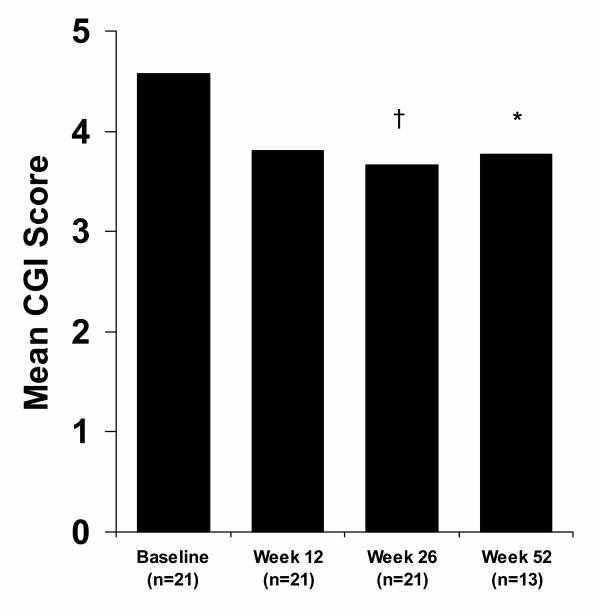
Mean CGI scores in patients with bipolar disorder followed up to Week 52. F = 6.92; ^†^p = 0.002; F = 4.91; *p = 0.036

In 21 patients whose symptoms were assessed at baseline and after 12 and 26 weeks of quetiapine therapy, significant (p = 0.002) improvements in mean CGI score were observed at Week 26 (Fig. 2). The improvement in mean CGI score remained significant (p = 0.036) in 13 patients who were evaluated after 52 weeks of treatment (Fig. 2).

### Safety

The most frequent side effects reported during quetiapine therapy were mild. These side effects were: dry mouth (4 patients; 19.0%), transient drowsiness (4 patients; 19.0%), sedation (3 patients; 14.3%), headache (2 patients; 9.5%), and constipation (2 patients; 9.5%). One patient discontinued quetiapine therapy in the second week of the study due to hypotension and drowsiness. There were no incidents of tardive dyskinesia or EPS.

## Discussion

Patients with bipolar I disorder who have inadequate responses to standard pharmacological therapy remain a common problem, despite the wide array of psychotropic agents that may be utilized [20]. This open-label study has demonstrated that long-term therapy with quetiapine may benefit patients by improving global symptoms of bipolar disorder and reducing the rate of depressive relapses as well as manic/mixed relapses. In this study, treatment with quetiapine for 26–78 weeks of treatment was associated with significant improvements in relapse rates and symptoms that were achieved with 26 weeks of treatment in 21 patients and maintained for more than 52 weeks in a subset of 13 patients. These results, while in agreement with a recent large, randomized, double-blind study that established significant improvements in bipolar depressive symptoms [12] as well as several clinical trials showing efficacy for patients with symptoms of mania [11,15,16], also suggest that the treatment effect observed with quetiapine is maintained in the long-term.

For each patient, long-term quetiapine treatment was added to ongoing medication, which included several classes of psychotropic therapies, such as traditional mood stabilizers and anticonvulsants, typical antipsychotic agents, and atypical antipsychotic agents. While polypharmacy with several kinds of psychotropic therapies is common in adults and children with bipolar disorder in the community [5,21], systematic studies of long-term antipsychotic therapy have generally used only traditional mood stabilizers as adjunctive therapy [22-25]. This study showed that adding quetiapine to a variety of ongoing therapies was well tolerated and associated with a low incidence of adverse events over the long term.

Although the cause is unknown, several studies have suggested an increased rate of antipsychotic-induced tardive dyskinesia and extrapyramidal symptoms (EPS) in patients with affective disorders compared with patients with schizophrenia [26-29]. Of particular importance for patients with affective disorders, no increase in tardive dyskinesia or EPS was reported during quetiapine therapy in this study.

Similar results have been reported in a small, open-label, 88-week study of 10 adolescents with schizoaffective disorder or bipolar disorder [30], indicating that quetiapine treatment is effective and well tolerated for long-term treatment in this population as well.

This study has a number of limitations, including small sample number and lack of a comparator. Furthermore, the design of an open-label study does not include randomization techniques and blinding of investigators to patient status, which may introduce selection bias. However, albeit a different study population, similar observations with quetiapine were reported in a study of comparable design [30]. Nevertheless, large, randomized, double-blind, placebo-controlled studies are needed to explore the benefits of adding long-term quetiapine to standard therapies for bipolar disorder.

## Competing interests

The author(s) declare that they have no competing interests.

## Authors' contributions

MCH, MGC conceived of the study, participated in the design of the study, coordinated the study, performed the statistical analysis and drafted the manuscript. AG, BC, JRC participated in its design and coordination. All authors read and approved the final manuscript.
